# Press discourses on ecological crises in the UK, Israel, and Hungary

**DOI:** 10.3389/fsoc.2023.1186410

**Published:** 2023-07-26

**Authors:** Nira Yuval-Davis, Isabel Meier, Rolly Rosen, Viktor Varjú

**Affiliations:** ^1^Research Centre on Migration, Refugees and Belonging, University of East London, London, United Kingdom; ^2^Department of Geography and Environmental Sciences, Northumbria University, Newcastle, United Kingdom; ^3^Political Science Department, University of Haifa, Haifa, Israel; ^4^Transdanubian Research Department, Institute for Regional Studies, KRTK, Pécs, Hungary; ^5^VFGI, Institute of Rural Development and Sustainable Economy (MATE), Kaposvár, Hungary; ^6^Institute of Social Connections (TKI), Faculty of Humanities and Social Sciences, University of Pécs, Pécs, Hungary

**Keywords:** ecological crisis, politics of belonging, press discourses, eco-relationalities, spatialities, temporalities, climate justice

## Abstract

This article explores the relationships between political projects of belonging and approaches to environmental and climate ecological crises via comparing centre-right and centre-left newspapers in the UK, Israel and Hungary. Our theoretical framework draws on Nira Yuval-Davis's work on the politics of belonging as a way of understanding and framing the different political projects that accompany reporting on ecological issues. Focusing on selected national and international case studies on these issues at the centre of public debate during the last two decades, the paper explores and compares these relationships by examining the eco-relational, spatial, temporal and normative framing dimensions of the political projects of belonging as expressed in these articles. The findings of the analysis show that, despite different cultural and historical contexts, the most significant dividing line is not among countries but between the different political projects of belonging of the newspapers. This can be seen even when dealing with country-specific, rather than international, case studies. Overall, centre-right newspapers tend to focus on narrow nationalist interests concerning the climate crisis and do not produce discourses of urgency to resolve the crisis except when reporting on major international political agendas. They are also more inclined to focus on the economic aspects of such efforts and how they would affect the “people”. The centre-left press, on the other hand, tends to prioritise ecological issues much more; it has wider global and planetary interdependent constructions of belonging and engages in the production of discourses of urgency in an attempt to solve the crisis and avoid future catastrophes. However, even in the centre-left press, especially in the UK, a tendency to remain within a western-centric perspective was observed.

## Introduction

This article compares attitudes to environmental and climate changes in centre-right and centre-left newspapers in the UK, Israel and Hungary. It is a part of a larger research project^1^ arguing that such attitudes are inextricably linked to different political projects of belonging (Yuval-Davis, 2012) and the “dirty work of boundary maintenance” (Favell, 1999).

The relationships people, societies and states have with their physical environments have, over the last two decades, come to occupy a major place in both formal and informal political agendas, at both local and international levels. These ecological concerns are driven by various disasters, both as a result of climate change as well as more localised factors, such as pollution of the land and sea in different corners of the globe, and have brought about a new sense of urgency (Dessler and Parson, 2019). A growing global protest movement is promoting the Green Agenda and pointing out the potential global dystopian outcomes if destructive ecological developments are not stopped and reversed. Indeed, increasing amounts of resources as well as legislation and policy on national levels have been directed towards environmental and climatic issues, enhanced by a series of international conferences, from the Rio UN Framework Convention in 1992 to the recent UN Climate Change Conference (COP27) in November 2022. While climate change denial is continuing to be an important political force, often enhanced by the power of international oil and energy companies, it is now increasingly accepted that there is an urgent need to reach international agreements on these issues and that they cannot be solved within national boundaries and policies.

However, the agendas of many of the green activist movements go beyond these issues. These movements also emphasise the disproportionate impacts of the climate crisis on the Global South, demanding a global response to the climate crisis that takes into consideration wider universal inequalities and histories of colonialism. They point to the link between these changes and growing global conflicts and pressures of displacement and asylum seeking in a world in which nativist and anti-migration lobbies have become stronger in many states. This normative political agenda has come to be known as the “climate justice” movement.

At the same time a wide range of other organisations and groups has turned instead to green nationalism (Conversi and Friis Hau, 2021), focusing on narrow nationalist ideologies as arguments against allocating major resources to control the Crisis more globally. While in Britain much of the debate relates to its imperial past (e.g., María and Macmillen Voskoboynik, 2021), in Israel the debate largely focuses on the particular features of Zionist settlement (e.g., Gorny, 2011), and in Hungary the environmentalist movement had a prominent role during the democratic transition as an opportunity to express general dissatisfaction with the communist regime (Mikecz and Oross, 2019, p. 139).

The diverse agendas of the different social and political agents who are engaged in ecological issues raise the question of what the relationships between these agendas and wider political agendas are. However, we also have another, more theoretical, reason to interrelate attitudes to ecological questions and people's politics of belonging.

In the literature, the ecological and climate crisis often tends to be analysed geologically—viewing it as a result of “the age of the Anthropocene” (e.g., Crutzen, 2006; Pulido, 2018), or historically—pinning down the destruction to “the age of the capitalocene” (e.g., Moore, 2016). The human species as a whole, or the capitalist system, are blamed for creating eschatological pressures which are “pushing the conditions of biospheric stability – climate and biodiversity above all – to the breaking point” (Moore, 2016, p. 1).

However, our approach is sociological as well as normative. We wish to highlight the differential attitudes as well as the contributions to this plenary transformation of different people in different and the same geographical locations and we therefore see our analysis as providing another, more nuanced, tool for the arguments of global justice.

The way this article approaches this task is by analysing press discourses on climate and environmental issues through specific case studies that were at the centre of international and national public debates during the last two decades. We agree with the Gramscian position that the mass media is a key site in which forms of knowledge struggle to achieve the status of “common sense” (Gramsci, 2011). This continues to be the case even in the age of social networks (Castells, 2015) although, given the growing influence of social media in setting up the political agenda, the press can be said to represent only what Grayson et al. (2022) call “the legitimate political spectrum”. The press plays a crucial role in the construction, reproduction and contestation of the ways major political issues are understood and related to specific political projects of belonging by, for example, controlling and constraining the visibility of particular groups (Edenborg, 2016).

## Political projects of belonging and climate crisis

Our theoretical framework draws on the politics of belonging (Yuval-Davis, 2012) as a way of understanding and framing the different political projects that accompany reporting on climate and environmental issues. Belonging is about emotional attachment, about feeling “at home”. And home, as Hage (1997, p. 103) has pointed out, is an “on-going project entailing a sense of hope for the future”. Part of this feeling of hope relates to home as a “safe” space (Ignatieff, 2001). In the daily reality of the early 21st century, since 2001 9/11 and the “global war on terrorism”; since the 2008 financial crisis and the “neo liberal double crisis of governability and governmentality (Yuval-Davis, 2012) the ongoing corona pandemic since early 2020, and the Ukraine war since February 2022, the emphasis on safety gets a new poignancy and complex new meanings to different people in different places on the globe. The growing ecological crisis, climate change and environmental erosion has added an urgent, sometime eschatological dimension to these anxieties.

While some scholars have attended to the ways that discourses on climate and environmental issues touch our feelings of safety and hope for the future (e.g., Ojala, 2012), not much attention has been given to how these discourses are always also related, explicitly or implicitly, to different competing political projects that construct specific collective boundaries as well as internal social divisions and power relations. Writings on green nationalism (e.g., Trafford, 2019; Conversi and Friis Hau, 2021) have illustrated how climate discourses can be used to further nationalistic projects, protecting and defending specific nations rather than focusing on different global impacts and inequalities.

This article explores the extent to which discourses on environmental and climate issues are primarily constructed as national or global discourses and whether the political centre-right (CR) and centre-left (CL) relate them to wider economic and societal issues. In order to do so, we draw on what Gellert (2005) and Bear (2017) and others call “socio-nature”—conceptualising society and nature as inseparable from each other and which therefore should not be analysed in abstraction from each other. Theorists arguing for the concept of socio-nature reject Marxist contentions that nature is a substratum to society, while also contending that nature is not merely a social construction. Post-dualist scholars (such as Haraway, 2006; Latour, 2014), but also Indigenous scholars (such as Deloria, 1999; Atleo, 2007) have dealt with non-human ontologies and nature's agency.

People's belonging and the politics of belonging are thus not abstract but embodied (Grosz, 2020). In this article we examine some of the major dimensions of this embodiment, their eco-relationality (ecological inclusionary boundaries); spatiality (spatial boundaries) and temporality (temporal boundaries) which any notion of belonging includes, explicitly or implicitly. We illustrate this via an examination of CR and CL press discourses on environmental and climate crises in three locations: the United Kingdom, Israel and Hungary. We also examine the normative dimension in these discourses.

We first examine discourses on ecological crises as these produce specific eco-relationalities. We approach relationality here as the extent and ways in which issues are framed in relationship to the ecological crisis as well as if nature and the non-human are included in or excluded from the collective “we” constructed in articles on relationship between people, society and politics. While many political projects of belonging naturalise land, flora and fauna as belonging to specific collectivities, their exclusion from discourses on ecological disasters construct them as external and lacking of agency.

Secondly, as Rowe (2005, p. 21) points out: “belongings are conditioned by our bodies and where they are placed on the globe”. The different contesting political projects of belonging in the media, therefore, also need to be analysed spatially. Here we are interested in exploring the ways these different discourses anchor themselves locally, nationally and/or globally, and how they draw national, racial and ethnic borders and boundaries related to specific constructions of both people and the planet (Yuval-Davis et al., 2019; Meier, 2020).

Thirdly, we explore what temporalities are produced through these discourses. We examine how the press discourses we studied relate not only to the time scales in which environmental climate crises are constructed, from a gradual process to an eschatological “end of the world” in 20 years rhetoric, but also as an intergenerational issue; whether environmental and, especially, climate crises, are referred to as primarily an issue for the younger generation and their concerned elders or rather as a crisis which is already affecting millions of people all over the world (Gonzalez, 2020; Sultana, 2022).

We also look at newspaper articles through the normative lens of wider global inequalities and power structures; i.e., focusing not only on the extent and location of the environmental and climate crises but their differential effects on different people within as well as between national and spatial boundaries. Here we are interested in learning whether and in what ways connections are being drawn between economic and social inequalities and the wider power structures causing them. These inequalities are part of wider global intersectional power structures that assign different value to human life as well as nature. They relate to the moral dimension of the politics of belonging which has come to be known as “climate justice” (Gonzalez, 2020; Sultana, 2022; Tilley et al., 2022).

## Local historical, legal, and political contexts

The history as well as the legal and political contexts of environmental concerns are different in the UK, Israel and Hungary. In the UK, with its intense industrialisation linked to its imperial past and class and race-determined social stratification, environmental policies developed out of earlier attempts of the aristocracy and higher classes to preserve natural beauty as national parks. Later, with the UK's transformation into a welfare state, policies developed aimed at conserving natural resources in the face of the destructive effects of industrialisation to the benefit of the whole of society. The latest phase of contemporary environmental policies is aimed at regulating the quality of air, water and land in the face of the growing dangers of the climate crisis (see, e.g., Johnson et al., 2010; Rollinson, 2010).

In Israel, where “modernisation” and industrialisation were tightly linked to the Israeli-Palestinian conflict, early Zionist ideology aimed at “making the desert bloom” and natural environments like swamps were seen as a challenge. The aim was to establish a modern agrarian and gradually more and more urban industrialised infrastructure instead of the more traditional Palestinian peasant and nomad economy that existed in the non-urban spaces in pre-1948 Palestine, when the state of Israel was established. These policies intensified after the establishment of the state, but their ecological and environmental cost have become clearer more and more over the years, so much so that certain policies, such as draining a lake (Hakhula) or chasing Bedouin goats out of fields had to be reversed (e.g., Hambright and Zohary, 1998).

In Hungary, industrialisation took place in the second industrial revolution under the Austro-Hungary Monarchy. After WWII, industrialisation was strongly influenced by Soviet style collectivisation. After the fall of the Berlin Wall in 1989 major political, economic and social changes took place in Hungary as well as in the rest of the ex-Communist bloc. The first environmental legislation to protect human lives was passed in the 1970s. In the post-communist period, with more international NGO and environmental legal and policy coordination, especially with the EU, more ecological policies and legislation were developed (Buzogány et al., 2022).

Despite all these sharp historical differences, environmental legislation in these three countries has become increasingly similar, especially since the 1990s, when environmental and climate issues came under the scrutiny of the UN and other international bodies. Neoliberal globalisation, the proliferation of international environmental and climate NGOs as well as other influences, including from the media, have also acted to reconfigure environmental and climate legislation, concern and debate in these three countries to resemble each other more. Another important factor is that in spite of very different political and economic histories, all three countries have been governed in recent years by right wing governments, which are simultaneously nationalist and neo-liberal.

## Methods and materials

The analysis in this paper and the research project as a whole is based on analysing the discourses in the different press articles using the situated intersectionality epistemological and methodological approach (Yuval-Davis, 2015; Yuval-Davis et al., 2019). It follows Collins' (1990) claim that one can approach the truth only by encompassing as many different situated gazes as possible. The truth of most realities is complex and multi-dimensional but not relativist. Only an encompassing perspective of intersected constructions of truth can help us in understanding certain social facts. A situated intersectionality approach, therefore, incorporates what McCall (2005) has viewed as two different types of methodological approach to study intersectionality, the inter-categorical approach, comparing the same variable in different contexts, and the intra-categorical approach, problematising understanding of one variable by different situated gazes in the same context. The epistemological challenge here is, therefore, not only to counterpose different vernacular realities vs. a multi-versal world (e.g. Latham, 2008), but also to highlight the differential perspectives of those who are differentially positioned in the same vernacular social encounter. For this reason, we examined newspapers in three different countries but looked at both mainstream centre-right (CR) and centre-left (CL) newspaper articles on the same or parallel issues.

We selected the following newspapers: in the UK, the Daily Mail (CR) and the Guardian (CL); in Israel, Israel Hayom (CR) and Ha'aretz (CL) and in Hungary, Magyar Hírlap (CR) and Népszava (CL). We then selected four international and three country-specific case studies: The international case studies being: (1) Trump leaving the 2015 Paris Agreement in June 2017, (2) The appearance of Greta Thunberg in the media and her UN speech in September 2019, (3) Biden re-joining the Paris Agreement in April 2021, and (4) the COP26 in Glasgow in November 2021.

For the country-specific case studies we chose instances of (1) floods, (2) heatwaves, and (3) pollution. In the UK, we selected the floods in the autumn and winter of 2019/2020 caused by exceptionally heavy rainfall and storms, the 2018 summer heatwave, and the 2020 death of 9-year-old Ella Adoo-Kissi-Debrah, the first death to be declared as caused by air pollution from the motorway near her home.

In Israel, we focused on the heatwaves that caused forest fires in 2019, the floods in Tel Aviv in winter 2020, and the pollution in the Evrona Nature Reserve in 2014 caused by a burst oil pipeline, which destroyed large parts of this important reserve in the south of the country.

In Hungary, we chose the heatwave of 2015, the flood at Lake Balaton in early 2018, and the plastic waste pollution of the Tisza River coming from beyond the country's border in 2020.

Four articles from each newspaper on each case study were included in the discourse analysis of the articles. When available, we chose two op-ed pieces and two news pieces. The articles were all published between 2014 and 2021. The criteria for selecting the articles were (1) presence of significant keywords in the articles' title, (2) clear relevance to the case study, (3) the length and detail of the article, and (4) the date published. The following information was extracted from the articles: (1) metadata, such as publication date and author, (2) article attributes such as timeline, what actors/voices/groups are included, and (3) thematic data, such as how events are described, who is responsible, who/what will suffer, questions of social inequality, economic, belongings and other political projects. The discursive thematic data was analysed using the 6-step thematic analysis technique (Braun and Clarke, 2021): (1) familiarising yourself with the dataset, (2) coding, (3) generating initial themes, (4) developing and reviewing themes, (5) defining and naming themes, (6) writing up.

## Results

We have organised our findings in four sections, following our theoretical framing in which ecological perspectives embody the perceived contexts of the different political projects of belonging, whether they are articulated explicitly or are just implicit in their discourses. As elaborated above, we differentiate between the eco-relational, spatial and temporal dimensions of these embodiments and have also added the normative dimension to link more explicitly our analysis to the ecological global justice perspective. Although the narratives in the articles that we studied can be seen as part of more than one analytical dimension, separating them in this way has enabled us to zoom in on each dimension. Obviously, given the selectivity of our case studies, these findings should not be received as complete. Many important articles relevant to our themes are not a part of our sample and thus are not included. However, we believe that what we found in the articles we analysed is conclusive enough, and we present a summing up table (Table 1) of our findings.

**Table 1:** Summary of the results.

**Summary of the results**	**CL vs. CR**	**UK**	**Israel**	**Hungary**
Eco-relational dimension	Diff. (CL vs. CR)	Centre right	Centre right	Centre right
- Some articles with total denial of the ecological crisis as a result of human impact on the environment in the CR press - Discredit of activists (by CR) - Ecological issues are excluded from the political projects of the CR - CR acknowledges environemnetal crisis by blaming political opponents - CR press tends to relate to the climate crisis as part of the national and international foreign affairs and reports on governmental activities	- CR dedicates much less space to cover env. and climate crisis - Discredit of activists (by CR) - Ecological issues are excluded from the political projects of the CR - CR acknowledges environmental crisis by blaming political opponents - CR press tends to relate to the climate crisis as part of the national and international foreign affairs and reports on governmental activities	- CR dedicates much less space to cover environmental and climate crisis - Some articles with total denial of the ecological crisis as a result of human impact on the environment in the CR press - Discredit of activists (by CR) - CR acknowledges environmental crisis by blaming political opponents - CR press tends to relate to the climate crisis as part of the national and international foreign affairs and reports on governmental activities
		Centre left	Centre left	Centre left
-Global socio-nature interdependence in a much more complex way than the CR *- includes international news and views in the agenda and does not only concentrate on the national-local point of view*	- CL: climate crisis high on agenda - Global socio-nature interdependence in a much more complex way than the CR *- includes international news and views in the agenda and does not only concentrate on the national-local point of view*	- CL: climate crisis high on agenda - Global socio-nature interdependence in a much more complex way than the CR *- includes international news and views in the agenda and does not only concentrate on the national-local point of view*
Spatial dimension	Diff. (CL vs. CR)	Centre right	Centre right	Centre right
- Extreme example of an exclusive national contextualisation - CR: Conflict between the interest of national economy vs. Paris Agreement	- CR tends to de-contextualise their descriptions of the specific ecological events - CR: No conflict between the interest of national economy vs. Paris Agreement	- CR tends to de-contextualise their descriptions of the specific ecological events - Extreme example of an exclusive national contextualisation
		Centre left		
- CR: use of personal pronouns, populist signifiers (us; we) vs. CL: “outside” terms (e.g., country)	- CR vs. CL presentation of national vs. regional effects of climate change. - Boundaries of belonging between the CR and CL → CR: nation, state and their various agencies; CL: also include scientists and NGOs - CR: use of personal pronouns, populist signifiers (us; we) vs. CL: “outside” terms (e.g., country)	- CR vs. CL presentation of national vs. regional effects of climate change. - Boundaries of belonging between the CR and CL → CR: nation, state and their various agencies; CL: also include scientists and NGOs - CR: use of personal pronouns, populist signifiers (us; we) vs. CL: “outside” terms (e.g., country)
**Temporal d**.	Diff. (CL vs. CR)	Centre right	Centre right	Centre right
- CR: Articles about No need for urgency (as well)	- In CR no historical presentations of climate crises	- In CR no historical presentations of climate crises - CR often tends to construct other socio
		- CR often tends to construct other socio-economic priorities as more urgent than those of climate change		-economic priorities as more urgent than those of climate change
	Similar. (CL vs. CR)	- Urgency for action (usually around COP) - Describe the severity (of cc impact), hence the urgency - Inter-generationality in the temporal discourse exists in both	- Urgency for action (usually around COP) - Describe the severity (of cc impact), hence the urgency - Inter-generationality in the temporal discourse exists in both	- Urgency for action (usually around COP) - Describe the severity (of cc impact), hence the urgency - Inter-generationality in the temporal discourse exists in both
Normative d.	Differences (CL vs. CR)	- Small size of country, thus their small contribution to the global problem is emphasised by CR - Echo of Trump's argument of PA leaving - CL: Emphasis of social inequalities	- Small size of country, thus their small contribution to the global problem is emphasised by CR - CR: Focus on the need to protect the nation from “racialized” others - CL: Emphasis of social inequalities	- Small size of country, thus their small contribution to the global problem is emphasised by CR - Echo of Trump's argument of PA leaving - CR: Focus on the need to protect the nation from “racialised” others - CL: Emphasis of social inequalities

###  The eco-relational dimension

Although most of the CR press in the countries concerned (especially in Israel and the UK), as well as all CL press, accepts that the global climate crisis is happening, there are significant differences in the ways in which this recognition is related to its political projects of belonging.

The CR press generally excludes and distances itself from incorporating climate and environmental crises into their political projects in several different ways.

First, while searching for case studies to focus on in our study, we found that overall, in the three countries, the CR press dedicates much less space to covering environmental and climate crisis issues than the CL press, thus excluding these issues or giving them low status overall in their political projects of belonging.

Secondly, in the CR press we found, at least in some of the UK and Hungarian cases, a total denial of the ecological crisis being a result of human impact on the environment. For example, the Daily Mail article of 26/7/2018 is titled “The predictable cry has gone up: climate change is causing the heatwave. Sorry, that's just hot air...” (Booker, 2018) and the Hungarian Magyar Hírlap states in an article that “The climate has always changed – there is no climate emergency” (Szarka, 2021).

In the British case, this denial is most often expressed through incoherent, contradictory and confusing claims. For example, Michael Howard, ex-leader of the UK Conservative Party wrote a by-line in the Daily Mail (Howard, 2018) titled: “30 years ago, Mrs Thatcher warned of man-made global warming. I fear this blazing summer is proving her right”. At the same time, he warns readers to “not rush to assume it is man-made climate change”. While calling overall for the UK public to listen to climate scientists, he is also claiming that the notion that climate is affected in any way by human activity is “often exaggerated”.

Such a devaluation of the climate crisis can be observed in the press narratives in a somewhat different way when attempts are made to discredit activists like Greta Thunberg. This is done via constructing her either as an innocent Asperger syndrome teenager who is “grotesque” (Galsai, 2019) or as suffering from normal “teenage outrage” (Vine, 2019), who has been used by others for “marketing” reasons (presumably marketing the climate crisis message) (Solomon, 2021).

In other articles, Greta Thunberg and other climate activists are described as “green militants” “fuelling a campaign to encourage thousands of children to skip school” (Borland et al., 2019). The Daily Mail criticises the attention that Friday for Future Climate actions received, labelling them as “playing truant”. They argue that “The most infuriating thing about last Friday's climate change school strike was not the tide of plastic left behind by protesters, or even the opportunistic rhetoric of politicians jumping on the bandwagon. No, it was the incessant virtue-signalling on social media of middle-class parents who had decided to accompany their little darlings on their inspirational day out, otherwise known as playing truant” (Daily Mail, 2019). In the Hungarian Magyar Hírlap an author poses the question: “Who should we listen to? To science or to the dilettantism of activists wrapped in humanist frills?” (Rab, 2019).

Even when not denying or devaluing the impact of environmental and climate disasters or the mass global protests against them, ecological issues are excluded from the political projects of the centre-right newspapers by depoliticising the discourse relating to ecological disasters. One mode of doing so, popular in the UK and Israeli CR tabloids, is by treating them as personal and familial tragedies without analysing the wider ecological factors involved. For example, when reporting on the death of nine-year old Ella, who died of air pollution in London, the UK's Daily Mail publishes an article entitled “Our heart-breaking last day together”, telling the story of how Ella's “tearful mother tells the air pollution inquest how she read Beethoven's love letters to her brilliant nine-year-old daughter hours before her death” (Bains, 2020). Or, when an Israeli couple died in a lift in Tel-Aviv as a result of flooding, a main article in Israel Hayom on the subject focused on the heart-breaking grief of their parents and family during the funeral (Cohen, 2020).

Another way of depoliticising the crisis that the CR press engages in, found especially in the Israeli press, is to treat specific instances of the crisis as natural, inevitable events that have nothing to do with political or policy issues. For example, when the huge crude oil spill in the Negev desert and the Evrona Nature Reserve occurred in December 2014, all the articles describing the disaster and its impact did not even speculate as to the possible social or economic causes of the leak (Lavi and Silberstein, 2014). Only in 2020, after the government published the results of the investigations showing that the leak was due to human error at the company, did such a report appear in the press, which was accompanied by the reassurance from the company that things have changed for the better since then (Silberstein, 2020). Similarly, when major fires spread all over the country in 2019, the exceptional high temperatures of the weather are mentioned in Israel Hayom, but no attempt is made to link the phenomenon to the global climate crisis (Diaz, 2019; Yalon, 2019).

When the CR press incorporates an acknowledgement of environmental crises into their political projects, they do it in two major ways. Firstly, by blaming political opponents. For example, Tozer (2019) focused on the error of the local Labour council as well as the Environmental Agency for reassuring the local residents the flood was not going to happen just a few hours before it actually did. In the case of the death of the couple who died in the lift during the flooding in Tel Aviv, the labour controlled local authority was blamed for the ineffectiveness of the emergency system (Roth-Avnery, 2020) and, in the Hungarian river pollution case, the blame was placed on the neighbouring countries (Magyar Hírlap, 2000; Pálfy, 2017).

Secondly, as the global climate crisis has been incorporated into the mainstream foreign policy agenda, especially around the annual COP meetings, the CR press tends to treat the climate crisis as a part of national and international foreign affairs and reports on governmental activities in this field, especially when important international meetings are taking place, often highlighting the importance of the contribution made by the prime ministers and presidents to the conference and the climate crisis agenda. This could be seen in articles in the UK's Daily Mail describing the leading role of the UK, under the leadership of Boris Johnson, in reducing greenhouse gas Emissions (Groves and Ellicott, 2021) and when Israeli PM Naftali Bennet boasted that Israeli high-tech companies can find the solution for such issues: “Israel is the ‘renewal of climate nation' and we're ready to lead the way” (Kahana, 2021). Similarly, in Hungary, president János Áder is quoted as emphasising that “Glasgow (COP26) is not only about raising ambitions, but is also about regaining credibility, taking action and seeing results as soon as possible” (Magyar Hírlap, 2021).

The CL press approaches these issues in a very different way. Overall, the subject is much higher on their agenda: the UK's Guardian even has a daily section dedicated to the subject, while Hungary's Népszava has a “Green Zone” column regularly reporting on issues related to ecology and climate, and Israel's Haaretz also has journalists specialising in these issues in particular. Moreover, they do not treat the subject merely as part of governance but as part of a much wider political agenda, in which NGOs as well as political parties, local authorities and national and international governments take part (e.g., Elekes, 2017; Leach, 2017; Reuters, 2017; Watts and Conolly, 2017). Above all, they highlight a global socio-nature interdependence in a much more complex way than the CR press. For example, when describing the meaning of the 2015 Paris agreement, which Trump had decided to leave, an article in Israel's Haaretz states:

“This is an agreement for the sake of planet Earth. For everything which grows and lives and breathes on this globe which we all share, with growing and eroding density” (Medav, 2017).

In another case, when reporting the oil spill in Evrona, Israel Hayom reported only on the immediate damage to the nature reserve and its animals, while Haaretz also explained the damage to the local insects, which are vital for plants, which, in turn, are needed to sustain larger animals (Rinat, 2014).

In a one-page interview article entitled “Earth: S.O.S.! – climate”, Hungary's Népszava cites the call made by the WHO and draws attention to the harmful effects of large-scale livestock farms around the world (Bihari, 2021).

###  The spatial dimension

In the previous section we focused on the different ways the CR and CL press have dealt with environmental and climate crises in terms of the extent and the ways they have excluded or incorporated these issues into their political projects of belonging.

In this section we examine the ways the different articles report and analyse issues relating to environmental and climate crises as a way of determining the boundaries of their imagined political community in which the CR and CL press are embedded. We contemplate the physical and social boundaries of the political projects of belonging which are assumed in their discourses. Once again, we found profound differences between the CR and CL press and the ways they narrate the issues. The CR press often tends to either ignore or de-contextualise their descriptions of specific ecological events. When they do contextualise them, they tend to see them through either nationalist or populist lenses. There are exceptions to this rule, which can be found, for example, in the Daily Mail article that links the local heatwave to the effects of global climate change (Fernandez, 2018) but this is somewhat unusual.

The CL press, on the other hand, tends to analyse these events as embedded within much wider boundaries. They view them not just as isolated, local, national or interstate events, but also as illustrations of global processes, highlighting the interdependence of all parts of the globe. As the Guardian article clearly states in its title “The weather in Britain is only a small part of a global pattern” (The Guardian, 2018). As such, their discourse embraces all levels, from local civic societies to transnational (rather than just international) social, political and ecological movements, from human life to all other forms of life on the planet.

An extreme example of an exclusive national contextualisation by the CR are the articles in the UK's Daily Mail and the Hungarian Magyar Hírlap that argue that global warming is actually good for the national interests of the UK and Hungary. “Global warming is not all bad... it'll boost UK tourism, says Bill Giles” (an ex-British weatherman) (Ruby and Ward, 2019). In the Hungarian case, a local economist wrote that global warming has potential benefit for local tourism (Bogár, 2017).

Another article in the Daily Mail highlights the exclusive focus on national boundaries in a different way. It states: “I couldn't give a monkey's about the double-standards of Joe Biden, Jeff Bezos, or any other of the preening global junketeers who turned up in Glasgow this week. But I am extremely concerned about the behaviour of our Prime Minister and his Cabinet, who increasingly behave as if the rules they impose upon on the rest of us don't apply to them.” (LittleJohn, 2021).

When discussing President Trump's decision to leave the Paris Agreement, we can find two different constructions of identification. On the one hand, there is detailed reporting of Trump's argument, which boils down to the conflict of interests between the people and workers who need jobs, unlike the elite (Daily Mail, 2017). On the other hand, there is a perceived potential conflict between the interest of the USA leaving the Paris Agreement and that of the individual countries included and analysed in this paper: “British taxpayers face paying more to help developing countries tackle climate change after Donald Trump's withdrawal from the Paris agreement left a £2.3 billion black hole” (Wilkinson and Groves, 2017), while Israel's Israel Hayom reassures its readership that Israel's continued support of the Paris Agreement helps, rather than damages, the Israeli economy (Hersch and Lavi, 2017).

While the CR tends to discuss the issues from a local and national perspective, the CL press narratives of belonging can be much more regional. For example, the Hungarian Magyar Hírlap tends to deal more with the domestic effects of heat waves (e.g., Csibra, 2015), while in its headlines Népszava (2015) tends to emphasise global effects: “Nobody is spared by the heat”, or regional effects: “European heat wave” (Hegyi, 2015). Similarly, Israel's Haaretz discusses the wave of extremely hot temperatures in terms of the whole Mediterranean zone (Hanin, 2021). Interestingly, in post-Brexit Britain, we found no such regional references in the articles we read in the Guardian but rather just the local/global perspective.

The construction of the boundaries of belonging and care affects the narratives of the newspaper articles even when not explicit. Thus, for example, when describing the wave of fires in Israel, Israel's Israel Hayom emphasised that the fires were not caused by the traditional fires lit on the Jewish holiday Lag BaOmer, which took place just before, but as a result of electric cables faults, thus protecting the Israeli Jewish, particularly religious population from being collectively blamed. On the other hand, in a sophisticated move trying to put the blame on possible Palestinian arson even without saying so directly, Israel Hayom (Yalon, 2019) placed the news item in an article that also described fires near the Gaza strip that erupted due to incendiary balloons sent over the border from Gaza, thus placing the fires within a clear context of the Israeli-Palestinian conflict, positioning Palestinians outside the boundaries of belonging. As a response, Haaretz included the Palestinians within the boundaries of belonging to the Israeli collective, protecting them from possible false blame, pointing out that police investigations had proved all such accusations false (Hasson, 2021).

The difference in the boundaries of belonging between the CR and CL press also involves which strata of the population is included in the collective “we” in the articles. One important source of difference relates to whose situated gazes are included in the articles. CR press articles tend to concern the nation, the state and its various agencies, international state leaders as well as directors and other representatives of economic corporations (Rácz, 2021). The CL press articles usually additionally include scientists as well as local, national and international NGOs (e.g., Medav, 2017; Watts and Conolly, 2017; Bihari, 2021).

While the CR press repeatedly uses the personal pronoun “us” and “we”, through which it articulates the local context as a homogenous national community with national interests (e.g., in the Hungarian Magyar Hírlap “What did we get in Glasgow”) (Rácz, 2021), the CL press tends to avoid using the personal pronoun “we”. Instead it tends to use the term “countries”, e.g., “pressure that countries need to feel” (Harvey, 2021) or “various countries have made loud promises about the protection of forests” (Horovitz, 2017; Rostoványi, 2021). However, in an article in Israel's Haaretz regarding Trump's decision to leave the Paris Agreement, Shlomo Avineri, a well-known political science professor, constructs the boundaries not just in geographic terms but also in terms of a universal political regime: “one can only hope that (Trump's) years in the White House will be over before too much damage is caused to the United States, to the world and to the future of liberal democracy” (Avineri, 2017).

Articles in the CR Daily Mail introduce the notion of “the people” as a boundary to the “ruling elite” (LittleJohn, 2021), who push through politics that are a threat to the general population. The CL press does not use the term “people” as a populist signifier but with different references to global and sometimes local populations or sub-populations (e.g., Carrington, 2021; Milman, 2021).

###  The temporal dimension

In the previous sections we discussed the eco-relational and spatial dimension of ecological crises to the political projects of belonging of the CR and CL newspapers we have been studying. In this section we focus on the temporal dimension of their political projects of belonging. Interestingly, especially in the CR publications, we found virtually no references to the historical developments that brought about the present national and global ecological crises. The point of departure tends to be the situation as it is at present (or, in the case of the international case studies, going back no further than the 2015 Paris Agreement). The discussion then refers to what needs to be done now or from now on.

We found that the temporal dimension in the political projects of the newspapers we have looked at is largely focused on the discourse of urgency (or lack of it). This sense of urgency has been produced in the press discourses through mainly two, not necessarily separate, climate and environment-related temporality narratives. The first one contests perceived present needs regarding imagined futures. As the related futuristic narratives are often eschatological, the more emphasis there is on them, the higher the sense of urgency expressed in the narrative. The second kind of temporality narrative is that of inter-generationality, i.e., through allocating either the roles of the main victims and/or the main bearers of the resistance to the coming global catastrophe to the young and future generations.

Once again, we found that in spite of local specificities the most important differences here also exist between the CR and CL narrative constructions. Although there is a general acceptance of the crisis, there are still some cases of climate denial in the CR press and even when there is an acceptance, to quote Michael Howard again the readers should “not rush to assume it (the crisis) is man-made climate change” (Howard, 2018).

Nevertheless, in the CR press there is some expression of the urgent need to take action to solve the climate crisis. This is manifested usually via quotes made by national prime ministers around the COP26 meeting, such as when the (Collins, 1990) quoted Boris Johnson in an article title: PM: “It's now or never on climate change” (2021). Similarly, in Israel Hayom quotes PM Naftali Bennet saying that “history will judge our generation's reaction to this threat” (Kahana, 2021), and in Hungary a Magyar Hírlap article reports the Deputy State Secretary responsible for youth affairs emphasising that “The essential part of the work...does not take place at the conference site...but...when we go home and put the...knowledge into practice” (Rácz, 2021).

The CL press, too, is full of expressions that describe the severity, hence the urgency, of the situation, with the use of terms such as “catastrophe,” “emergency,” “tipping point,” “dangerous,” “screaming at us” (e.g., Letters, 2019), “No one is spared by the heat” (2015) and of demands for action rooted in the present through the repeated use of the word “now” such as “radical changes are needed now” (Letters, 2019). “The ecological clock is ticking and they demand that we change our habits and lifestyles” (Barne'a, 2019).

The CR press often tends to construct other social and economic priorities as being more urgent than those of climate change even when there is no denialism. They widely reported Trump's argument when leaving the Paris Agreement that greening the economy is against workers' interests but even beyond that, the Daily Mail, for instance, argues that protecting the nation and its people needs to take priority over climate issues (LittleJohn, 2021). Similarly in Hungary, in Magyar Hírlap, a theologian author downgrades the importance of the “climate catastrophe” and writes that “We live in an idiotic world where groups are organised to protect the former (viz. environment), the postmodern man is sick with a thousand problems, of which the most important, the spiritual, are hardly offered a cure” (Kiss, 2020). In Israel the denial of the importance of the climate crisis by the CR press is not mentioned directly in the cited article, but instead they use more subtle ways, such as refraining from covering Thunberg's speeches almost altogether.

In addition, many right-wing newspaper articles use the conjunction “but” to create these discourses of non-urgency: “There is no doubt hers is a powerful, important message. But…”, “Climate change is undeniably a huge problem we must all face; but…” (Vine, 2019). Thus, the majority of CR articles were concerned with “protecting” the present generation, their perceived struggles, crises and the adverse economic impact of introducing radical green policies (e.g., Wilkinson and Groves, 2017).

The inter-generationality in the temporal discourse exists on both right and left. For example, the title of a Daily Mail article is “Act now for the generations yet unborn” (Ellicott, 2021). However, this is especially strong in the CL press. In an opinion piece from Horvath (2019), the Guardian reports “For this reason we have to understand this wave of children's disobedience (even skipping school) as an attempt to literally take back time (the future) from a system that is founded on extraction not only of natural resources, but of time and the future itself”.

In the Guardian, the Croatian philosopher Srecko Horvat claims that we are waging war against our children: “The first war against our youngest citizens is the destruction of the planet, which is the theft of their future; the second war is the overreaction to the current children's protest (Horvath, 2019). It reflects our western cultural tendency to simultaneously treat children like pets…The billionaire business leaders of Davos applauded the Swedish girl—, but from the outside it looked like a sideshow, a piece of diversion, rather than anything that would really cause them to change direction.”

In Haaretz, in an article entitled “Listen to the children” Amir Barnea states that “For the first time in many years, it is the youth who is leading the global struggle to a very large extent. It is difficult not to be impressed by young children, speaking with self-confidence and clarity, looking world leaders in their eyes” (Barne'a, 2019).

In the Guardian, however, George Monbiot warns that this is “a lot to dump on young people. But there are plenty of veterans, many of whom have far more experience than I do, who are ready to offer advice and help. Any support must come on the young strikers' terms: they lead, we follow. But they carry a terrible burden: this is a struggle they cannot afford to lose. We will help them lift it if they wish” (Monbiot, 2019).

###  The normative dimension

Until now we have examined the eco-relational, spatial and temporal dimensions of the ways discourses on environmental and climate crises have been constructed and embodied in the CR and CL political projects of belonging of the newspapers we've studied.

In this section we move from the descriptive and analytical discourses of the local and global ecological crises to the normative, which are sometimes explicit but also often implicit in the press discourses. Who is responsible for the crisis, what are the power relations involved, what kind of injustices, exploitations and other forms of social, economic and political inequalities have been involved and, of course, who should bear the cost of preventing it; in other words, what is the just solution to the crisis? In this way we take a step away from a preconceived generalisation that all humans living under the global capitalist system are to blame and examine the different perceptions of this issue in these newspaper articles.

As in our findings in the other sections, the construction of global and local power structures and inequalities differ significantly between the centre-right and centre-left viewpoints in the press we analysed, although the different histories and global positionings of the three countries play a somewhat larger role in this case. The relatively small size of the three countries and thus their relatively small contribution to the global problem is emphasised by the CR press of all countries, especially in the cases of Israel (Kahana, 2021) and Hungary (e.g., Bertha, 2021). Overall the British CR articles frame global agreements involving global climate justice issues as well the re-distribution of recourses as punishment for the “developed world” and a threat to “the people”: “While Britain has reiterated its support of the Paris Agreement, some British critics of the deal have also said it ‘penalises the industrial base of the developed world to the benefit of the economies of China and the rest of the emerging economies”' (Tough talk from a man who said global warming is Chinese hoax, 2017).

In the same vein, the coverage of Trump's decision to leave the Paris Agreement in the CR newspapers tends to echo Trump's argument that the Paris Accords will deprive thousands of workers of their jobs and injure national interests (e.g., Fernandez, 2017), and the Hungarian Magyar Hírlap echoes Trump's claim of the USA being a victim of the Paris Agreement (e.g., Ulicza, 2017).

In Israel, Israel Hayom presents its scepticism regarding the Glasgow Conference's decisions relating to global justice by putting the decision inside quotation marks. Thus, the subtitle of the article summarising the conference's decision reads “It was decided to ‘reduce' the use of coal, and not to completely remove it from use for electricity production - due to pressure from India and Iran. In the third and final draft, the rich countries are called to ‘double by 2025' their support to developing countries” (Lin, 2021).

CR newspapers focus on the need to protect the nation from racialised others, other countries, as well as the “ruling elite” (e.g., LittleJohn, 2021). In the Hungarian case, a contrast is drawn between the EU and Hungary, as representing liberal vs. nationalist world views. In the Hungarian centre-right newspapers, which often echo Orban's anti-liberal rhetoric, the two sides are sometimes presented as David vs. Goliath, i.e., as two communities with unequal forces being at war with each other. In Magyar Hírlap, the anti-Brussels narrative, adopted by the paper in recent years, is often repeated. In the op-ed article related to COP26, the author writes, for example, that “Brussels...by increasing utility costs, would transfer the negative effects of climate protection to European, including Hungarian, taxpayers” (Bertha, 2021). Israel's complex position within this, not being part of the Western history of colonisation as a state, yet practising a different variation of colonialism in the present, obviously influences the media's position regarding these issues and the CR press takes the position of detached observer: “The most serious dispute between the parties was whether rich countries in the developed world should be required to establish an official fund to help the poorer countries cope with the effects of the climate crisis” (Lin, 2021).

Global as well as national civil society organisations also play important part for the CL press (e.g., Horovitz, 2017; Rinat, 2017; Varga, 2019). These organisations articulate global social inequalities in relationship to the ongoing effects of the power of big global corporations. This difference in reporting in relation to social inequalities and wider global power structures becomes particularly apparent in the case study of COP-26 as well in the Paris Agreement reporting. Most CL newspapers reporting on the COP-26 meeting in November 2021 put emphasis on the disproportional burden the global South has to carry re the effects of the global climate crisis in at least some of their articles. A Guardian article (Figueres, 2021) describes “the shocking escalation of impacts being felt by the most vulnerable”, and the paper also adds that “the most acutely felt, glaring void is the lack of support for the most vulnerable nations to cope with quickly accelerating loss of homes and livelihoods and damage to lands and infrastructure”. An article in Haaretz by Dov Hanin, a left-wing and climate change activist and ex-MP states that: “Again we saw in Glasgow the power of the coal, oil and gas corporations that fought in all manners to prevent an effective resolution (Hanin, 2021). But we also saw the huge public recruitment, especially of young people, for life and the necessary change”. The Hungarian press cited the words of the president of Palau island, pointing out the vulnerability of developing countries and islands (Rostoványi, 2021). It cites Tuvalu's leader's words about the negative impact the islands have to face and addresses the inequality of CO2 emission and its impact globally (Varga, 2021).

It is important to note, however, that rather than engaging with narratives of climate justice and the colonial histories, which position these countries very differently in terms of effects, agencies and political power, former colonies and other Global South countries are labelled in the CL press as “developing countries”, “most vulnerable countries” as well as “poorest nations”. Even when describing the conflict in COP26 and India's and China's influence in the language of the resolution being watered down from “phasing out” to “phasing down” (Carrington, 2021) they, too, do not contextualise these conflicts through wider global power structures and longstanding social inequalities. Rather, there is a satisfaction with even the limited progress that has been done: e.g., Haaretz argues: “Some progress has also been made in establishing a mechanism for compensating developing countries by rich countries for damage and loss of life and property as a result of the effects of the climate crisis… The very decision in principle that it is appropriate to establish such a payment, is already progress” (Rinat, 2021). The discourse here is on the unequal but natural differential effects of the climate crisis rather than on the effect of explicit political and economic global policies which need to be comprehensively changed before any real global climate justice can be achieved.

## 6. Conclusion

The article develops a sociological situated intersectional approach for understanding the links between different political projects of belonging and attitudes and positions regarding the current ecological and climate crisis. We have illustrated some of the ways different situated gazes and political perspectives of different CR and CL newspapers in the UK, Israel and Hungary have reported and discussed different ecological related national and international events in our socio-nature.

As mentioned in the methodological introduction, we incorporated in our approach both inter- and intra-categorical comparison. What we found is that in the case of mainstream right and left press in the UK, Israel, and Hungary in the last two decades, the constructions of political discourses on ecological issues are very similar while the intra-variations of meanings in each country are much smaller. However, this does not mean that the CL and especially the CR positions are necessarily homogenous and consistent. Differences in reporting were observed, within the same newspaper in articles relating to the same environment or climate related events.

The overall similarity between the CR and CL ecological perspectives has proven to be the case not only when analysing our international case studies, when newspapers in different countries are often fed the same information from international news agencies such as Reuters, but also when we examined different national environmental crises in each country.

We show how the different dimensions of belonging, eco-relational, spatial, temporal, contextualise (explicitly or not) the ecological discourses in the articles we examined as well as their normative stance. We show how the CR and CL tackle in different ways “the dirty work of boundary maintenance” central to different political projects of belonging, because of their different perceptions of the extent and nature of these boundaries.

Most significantly, we found that CR newspapers perceive ecological issues in a narrower relational way than the CL press. Overall, they pay less attention to a subject that does not seem to affect their everyday concerns, even when they do not deny its effects altogether—something which, by now, is quite rare, even in the CR press, with the probably partial exception of Hungary. When they do deal with ecological issues they tend to focus on personal stories and local perspectives in a depoliticised way. Environmental events are often portrayed in these newspapers as inevitable “natural disasters”, and newspapers rarely present human responsibility for these events, with the possible exception of local authorities governed by CL parties. CL-oriented newspapers, on the other hand, present the ecological events, national as well as international, as being related to broader contexts, planetary as well as national and local. They give expression to a greater variety of voices in relation to the issue in question and tend to offer structural solutions to prevent the recurrence of such events. One might get the impression, reading CR press, that to a large extent the whole notion of climate crisis is a product of left-oriented political imagination. At the same time, paradoxically, it is also a major point on the agenda not only in the international political scene, but also in the reconstruction/expansion of the global market, which climate activists tend to call “green-washing”.

CR oriented newspapers construct clearer boundaries of separation, both between humans and nature (which is perceived as uncontrollable) and between the country's nation and the rest of the world (who are of secondary importance in the debate). On the other hand, CL newspapers tend to see connections, both between humans and nature and between the inhabitants of the whole world and themselves. Such relational connections are economic, political and moral in terms of mutual responsibility. We found such connections not just spatially but also temporally, in terms of the responsibility of the present generation to secure the welfare or even the possibility of the survival of future generations. Anxiety about the future is often much more prevalent in the CL press than in the CR.

However, due to their different colonial histories and also their size, there is a difference between the three countries regarding the issue of “climate justice”. In Britain the CL press is much more concerned than the CR press, not just about the overall adversarial effect of the global and local ecological crisis, but also about what has come to be known as “climate justice”; the differential impact in both creating and suffering from the adverse effects of environmental and climate disasters. For different complex historical positionings and political reasons, this is not the case in Israel and especially not in Hungary.

If belonging, as mentioned at the beginning of the paper, is related to a process of feeling happy and safe at home, discourses on the left are likely to produce more (climate) anxiety, depression and even rage not as a pathological but as a reasonable and healthy response to an existential threat. However, these discourses also produce feelings of connection and of feeling at home through their focus on our interconnectedness/inter-being with nature and care for more equal and just distribution of power and conditions of individual and collective existence on the globe. So much so that for the first time, following discussions in COP26, it has been decided to put “damages and losses” on the formal agenda of the COP27 conference as a result of the uneven exploitation of natural resources.

In a world struggling with the aftereffects of the COVID-19 pandemic, a reconstitution of agonistic world powers intensified by the war in Ukraine, a spreading global systemic crisis of governability and governmentality due to intended and unintended outcomes of deregulated neo-liberal globalisation, political projects of belonging, willingly or reluctantly, have to confront the physical and ecological context of their social, economic and political lives. We see this article as a contribution to understanding this relationship as well as to the growing global ecological justice movement. The paper points to the limits of a generalised discussion on the age of the Antropocene and even of the Capitalocene to accurately capture the nuances of issues of inequalities and justice and illustrates the contribution that can be made through approaching the entanglements of political projects of belonging with ecology.

## Data availability statement

Publicly available datasets were analysed in this study. This data can be found here: Newspapers article relating to the UK and Israel are publicly available on the newspapers' website. Relating to the Hungarian case, the Arcanum digital repository was used: https://www.arcanum.com/en/.

## Author contributions

NY-D and IM contributed to conception. N-YD wrote the first draft of the manuscript. IM, RR, and VV wrote sections of the manuscript. All authors contributed to collecting articles, performing the analysis, and to the design of the study. All authors contributed to revising the manuscript, have read, and approved the submitted version.

## Footnotes

^1^Nira Yuval-Davis' Emeritus Research Felloiwship EM-2020-069: Climate Change and the Politics of Belonging
